# Deaths from Chloroform

**Published:** 1865

**Authors:** 


Deaths from Chloroform.—An inquest was held on Friday, June 24th, at King's College Hospital, on the body of Mrs. E. Ruth, aged 29, who died from the effects of the vapor of chloroform during an operation for removing a tumor in the urethra. A question arose as to the quantity administered to her on a former occasion, and the absence of books of reference in such cases in the hospital. The following verdict was returned:—"That the deceased died from the effects of the vapor of chloroform," and the jury expressed their opinion that proper case-books for reference should be kept in the hospital. Another fatal case occurred at Middlesex Hospital, on Tuesday, July 5th. This was an operation for the removal of a large tumor from the face, during which the patient died on the operating table. A third case occurred at St. Mary's Hospital, where an inquest was held on Monday, July 18th, on the body of James Birch, aged 56, who died while under the influence of chloroform, administered previous to an operation for the removal of the bone of the great toe. A *post mortem* showed extensive disease of the heart.—*London Phar. Jour.*

